# Hot Topics in Clinical Oral Implants Research: Recent Trends in Literature Coverage

**DOI:** 10.3390/dj4020013

**Published:** 2016-05-24

**Authors:** Vesela Valkova, Ceeneena Ubaidha Maheen, Bernhard Pommer, Xiaohui Rausch-Fan, Rudolf Seemann

**Affiliations:** 1 Medical University of Vienna, Bernhard Gottlieb School of Dentistry, Sensengasse 2a, A-1090 Vienna, Austria; vesela.e.valkova@gmail.com (V.V.); ceeneena@yahoo.com (C.U.M.); 2 Academy for Oral Implantology, Lazarettgasse 19/DG, A-1090 Vienna, Austria; 3 Medical University of Vienna, Rausch-Fan Laboratory, Sensengasse 2a, A-1090 Vienna, Austria; xiaohui.rausch-fan@meduniwien.ac.at; 4 Medical University of Vienna, Department of Oral and Maxillofacial Surgery, Währinger Gürtel 18-20, A-1090 Vienna, Austria; rudolf.seemann@meduniwien.ac.at

**Keywords:** immediate implant loading, guided implant surgery, minimally invasive techniques, immediate implant placement

## Abstract

This systematic review looks at thematic trends in clinical research publications on dental implants. For this purpose, MEDLINE electronic searches as well as additional hand searches of six main journals in the field were conducted. A total of 2875 clinical studies published between 2001 and 2012 met the inclusion criteria and were subjected to statistical analysis. Hot topics in dental implant literature included immediate loading (14.3%), bone substitutes (11.6%), cross-arch full bridges (8.0%), and immediate implant placement (7.5%). A significant increase in scientific interest for immediate loading (+6.3%, *p* = 0.001), platform switching (+2.9%, *p* = 0.001), guided implant surgery (+1.9%, *p* = 0.011), growth factors (*p* = 0.014, +1.4%), piezoelectric surgery (+1.3%, *p* = 0.015), and restorative materials (+0.7%, *p* = 0.011) was found. A declining scientific interest in onlay grafting (−0.3%, *p* = 0.042) was recorded. The findings regarding current clinical oral implants research tie in with better-informed consumers and increased patient demands. Our results demonstrate an increasing interest in techniques that avoid complicated procedures such as bone grafting and that reduce treatment duration.

## 1. Introduction

The present special issue of *Dentistry Journal* deals with “Advances in Implant Dentistry,” and the following keywords denote hot topics in this field: template-guided implant placement, minimally invasive techniques, short lengths and reduced implant diameters, novel bone grafting techniques, medically compromised patients, peri-implantitis treatment, immediate placement and restoration, transition from a failing dentition, CAD/CAM prosthetics, and optical intraoral impressions. As the first paper in this special issue, the following review aims to provide the background to recent trends and “hot topics” in advanced and minimally invasive oral implant treatment [1].

The concept of osseointegration of oral implants was introduced by Branemark 40 years ago and set the precedent for new knowledge in oral medicine. Since then, oral implantology has become one of the most investigated topics in dental medicine, with exponential growth in the use of implant products [2]. Data shows that the number of implants used for oral rehabilitation in the USA increased ten-fold between 1983 and 2002 and also ten-fold from 2000 to 2010 [3]. While previously the primary aim of research on oral implantology was to find ways to rehabilitate function [4], many efforts nowadays are focused on the shortening of treatment procedures, simplifying surgical techniques, and esthetic improvement [5]. It is well known that oral implantology is a prosthetically driven field with a major surgical component [6]. Therefore, the current state of the art in implant dentistry represents advances in both surgical and prosthodontic techniques [5].

Keeping pace with research development, the aim of this systematic review was to investigate contemporary issues in oral implantology research and to perform a topical trend analysis of clinical studies published in the time period from 2001 to 2012.

## 2. Material and Methods

### 2.1. Search Strategy

A MEDLINE electronic literature search was conducted, limited to clinical studies on dental implants published between 2001 and 2012. The search term “dental implant,” sorted by “year of publication” was used in order to capture all relevant articles [7]. Additional hand searching was performed to examine six main journals in the field: *The International Journal of Maxillofacial Implants*, *Journal of Oral Implantology*, *Clinical Oral Implant Related Research*, *Implant Dentistry*, *European Journal of Oral Implantology*, and *Clinical Oral Implant Research*. Two reviewers independently identified all trials [8]. The PubMed search initially identified 15,695 publications, and 5048 additional results were identified by hand search. These studies were screened for their relevance based upon a threshold set [9]:

- inclusion criteria: prospective and retrospective studies, cross-sectional studies, case-control studies, case reports with at least 10 patients
- exclusion criteria: non-English publications, statistical studies, animal studies, finite element analyses, *in vitro* studies, review articles, and case series with fewer than 10 patients.

A total number of 3695 articles were subjected to abstract review. Where the abstract provided little information, a full text analysis was performed. Authors of potentially relevant publications, which were not available or lacked data, were contacted and asked for cooperation. Ultimately, 2875 clinical studies were identified as meeting the inclusion criteria. Our goal was to investigate how trends change over time as regards the topics examined in modern implant dentistry research. In this respect, we have determined that 31 topics were appropriate: 23 of them concerned surgical issues and 8 dealt with prosthodontic issues (Table 1). First, all relevant publications were screened for the topics listed in Table 1 independently by two reviewers. Thereafter, the results were verified, and all doubtful publications were discussed before the final decision was taken.

### 2.2. Statistical Analysis

As mentioned above, 2875 publications were analyzed. In order to find statistical trends in respect to the relevant topics between 2001 and 2012, Poisson regression analysis was performed, taking the level of significance as *p* ≤ 0.05, using R-project statistical software version 3.1.0. This statistical test was used to model count data, which in this case was the number of publications. *p*-values were calculated for every topic, taking into account the relative number publications per topic from the total number of publications.

## 3. Results

The surgical and prosthodontic topics of interest were computed as percentages of the total number of publications (Table 1). Among the most covered surgical topics in the literature were immediate loading (14.3%), bone substitutes (11.6%), immediate implant placement (7.5%), simultaneous implant placement with bone augmentation (6.4%), onlay grafting (4.3%), medically compromised patients (4.0%), healing modality (3.7%), transcrestal sinus floor elevation (3.0%), flapless surgery (2.7%), socket grafting (2.6%), and guided surgery (2.4%). Immediate loading (14.3%), cross-arch implant bridges (8.0%), early loading (4.5%), and platform switching (1.7%) were ranked as the most prevalent prosthodontic issues in current oral implant research.

**Figure 1:**
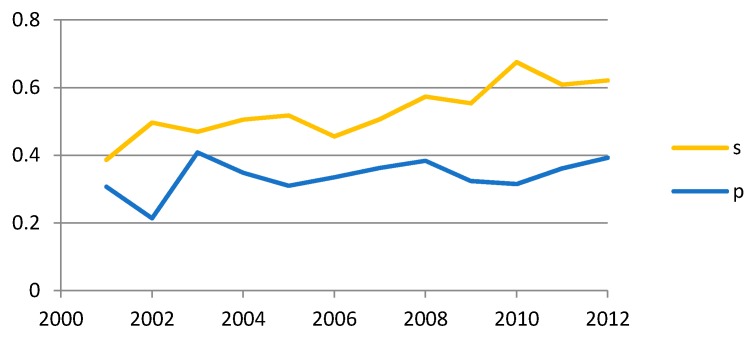
Literature coverage of surgical (s) *versus* prosthodontic (p) issues: x-axis indicates year of publication, y-axis indicates the ratio of numbers of publications (surgical/prosthodontic) to the total number of publications per year.

The surgical issues were the more prevalent topics, demonstrating an increasing rate of publications over the time in terms of mean coverage (0.53 ± 0.01) per publication (Figure 1), as compared to prosthodontic issues (0.33 ± 0.05 hits). The mean coverage values were estimated based on yearly ratios: the number of prosthodontic/surgical publications per year in relation to the total number of publications per year. The significant increase in publications on surgical issues over the years was demonstrated by Poisson regression analysis (*p* = 0.002).

**Figure 2:**
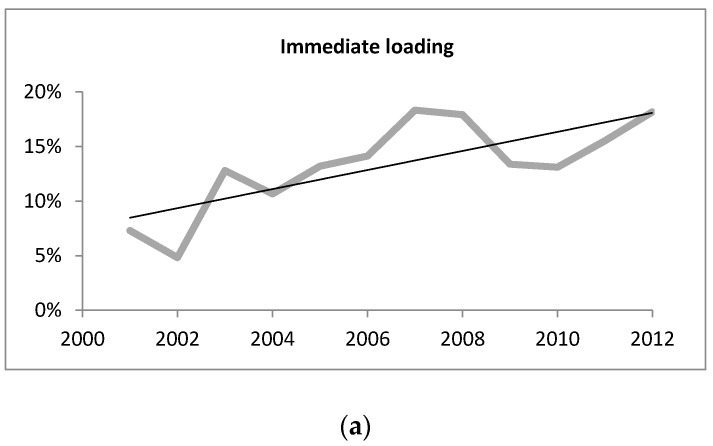

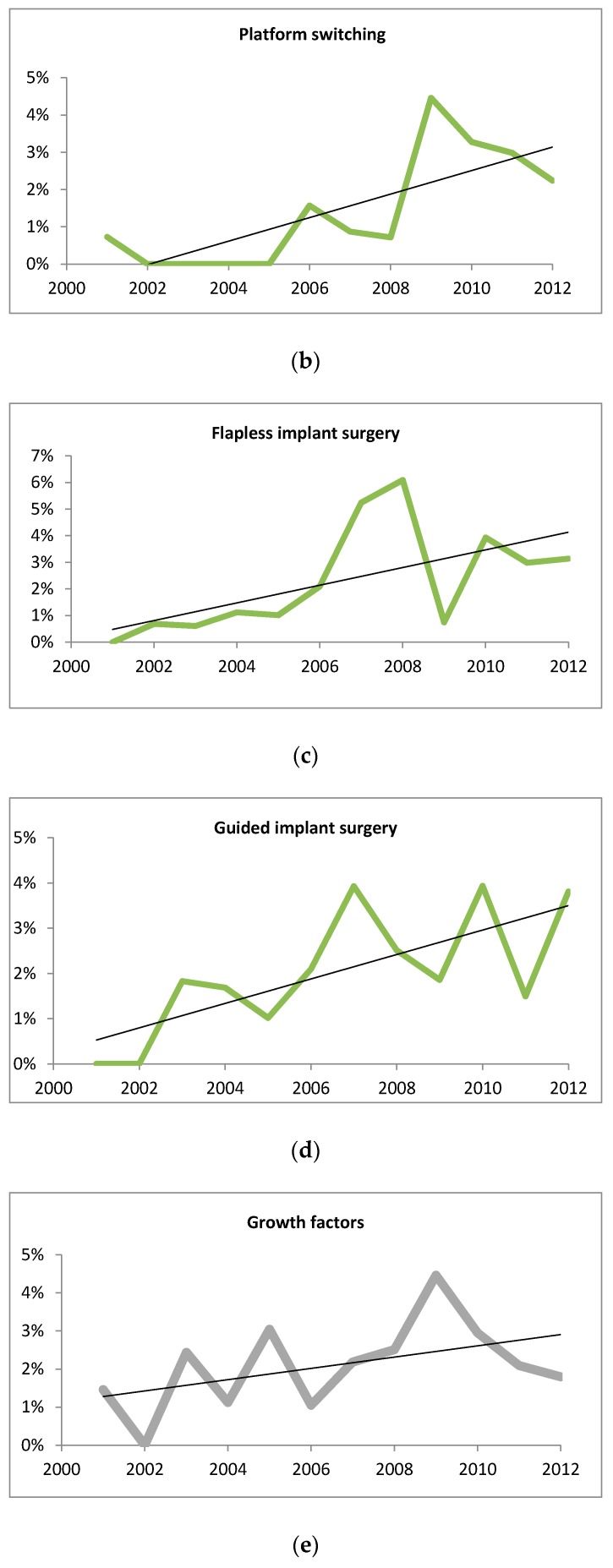

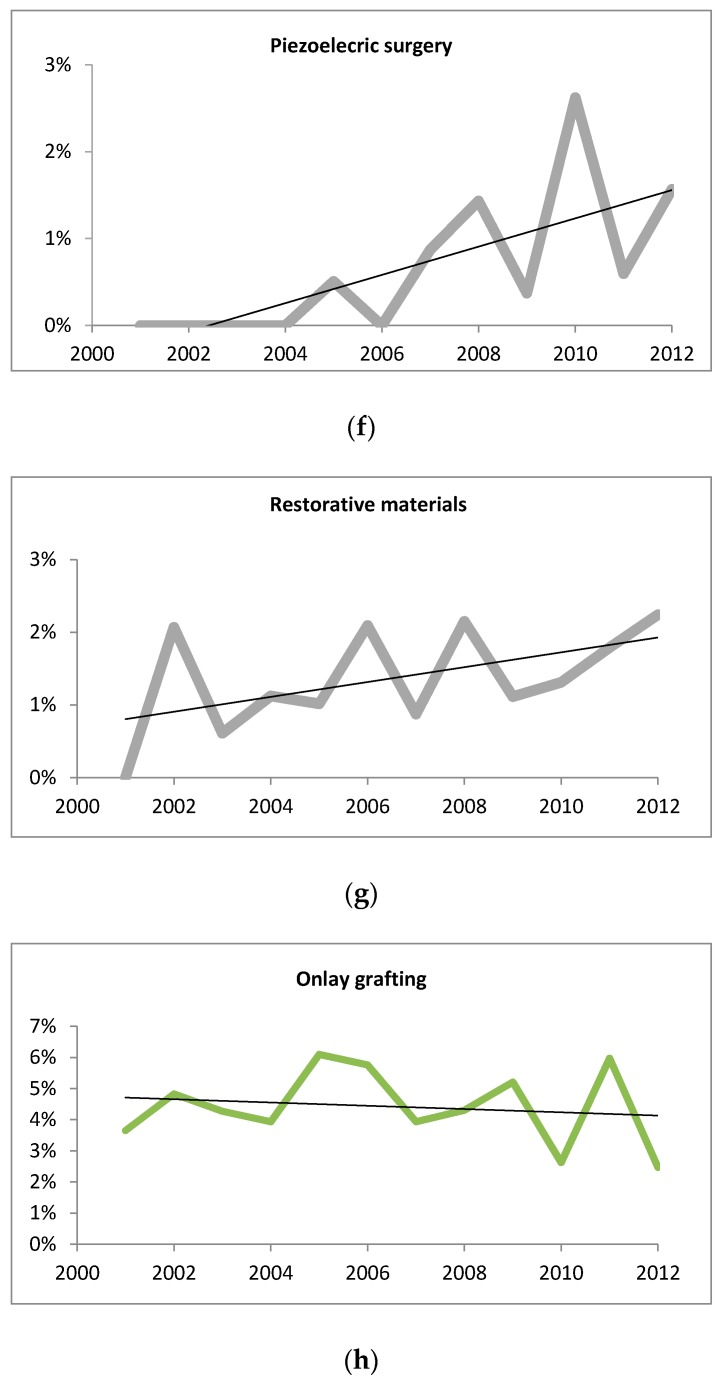
Trend curves (percentage out of the total number of publications per year) for (**a**) immediate loading; (**b**) platform switching; (**c**) flapless implant surgery; (**d**) guided implant surgery; (**e**) growth factors; (**f**) piezoelectric surgery; (**g**)restorative materials; and (**h**) onlay grafting.

A total of eight topics showed significant trends (*p* < 0.05) over the years 2001 to 2012 (Table 2). Immediate loading demonstrated the highest increase with a positive change of +6.3% and *p* = 0.001 (Figure 2a). Platform switching (+2.9%, *p* = 0.001) was the second topic showing a significant increase; however, only one relevant article was detected between 2001 and 2006 (Figure 2b). These topics were followed by guided implant surgery (+1.9%, *p* = 0.011), growth factors (+1.4%, *p* = 0.014), piezoelectric surgery (+1.3%, *p* = 0.015), and restorative materials (+0.7%, *p* = 0.011). The green line represents the percentage of the total number of publications for every year. The black trend line reveals the relationship between the year of publication (x-variable) and the percentage of the total number of publications (y-variable). Since there were no publications on platform switching between 2002 and 2005, there is a negative trend line intercept starting from 2001 (Figure 2b). Decreasing scientific interest and a corresponding downward trend were recorded for the topic onlay grafting (−0.3%, *p* = 0.042).

## 4. Discussion

Comparisons of published clinical trials per year revealed a trend of increasing interest in conducting clinical trials, starting with 137 relevant articles in the year 2001 and reaching the number of 446 publications in the year 2012. However, even the total number of 3695 articles is smaller than the total number of 4655 clinical studies published between 1989 and 1999 reported by Russo *et al.* [10]. Given that the number of publications increased with every year, it was considered more appropriate to perform Poisson regression analysis related to percentage-based values rather than related to absolute values for all topics.

Immediate loading proved to be the most studied topic in the last decade (Figure 2a). This avid scientific interest can be explained by several advantages it offers, such as shortened treatment protocols, immediate rehabilitation of the function, and high patient satisfaction. Meta-analyses on single-tooth implant placement have shown encouraging results for the immediate loading protocol as a promising alternative to conventional loading, as it may be equally successful and may not significantly affect marginal bone resorption and implant success rates [11,12,13]. Another meta-analysis by Papaspyridakos *et al.* [14] reported that there was no significant difference between immediate, early, and conventional loading in edentulous patients with fixed prostheses, and all three protocols showed a high level of success. However, other reviewers disagree with this assessment of the unimpaired success of the immediate loading protocol. A meta-analysis of clinical studies comparing the immediate and conventional loading of single tooth implants discovered that immediate loading has a significantly higher risk of implant failure [15]. Schimmel and coworkers [16] concluded that, despite the high implant survival rates, the conventional and early loading protocols are superior to immediate loading as better documented protocols, providing better results in the first year of loading. A survey among implantologists from 16 countries all over the world stated that immediate loading was the treatment protocol most accepted by dentists in Australia and Europe [17]. Based on these controversial statements in the literature, it can be concluded that there is still a lack of well-designed RCTs concerning loading protocol [18] and immediate loading may well retain its place as a hot topic of discussion over the coming years.

The platform-switching concept arose in 1980 with the introduction of the wide diameter implants. Due to the lack of commercially available matching components for wide diameter implants, the standard-diameter abutments were used. Later, it was found that “platform-switched” implants demonstrated osseointegration with less initial crestal bone loss and were thus superior to the “platform-matched implants” [19]. However, the first introduction of this concept appeared in 2005 [20]. Radiographic observation over a period of 13 years demonstrated that platform switching resulted in little or no crestal bone loss as compared to the conventional implants, whereas marginal bone resorption of 1.5 mm on average was accepted as one of the criteria for success of the dental implant [21]. Our study shows that the increasing publication rate of clinical studies happened to coincide with the first official introduction of this concept, with a positive linear trend for this topic starting in 2005 (Figure 2b). Since guided surgery is performed in combination with the flapless procedure in most cases, [22] the similarity in literature coverage, illustrated in both scatter plots, does not come as a surprise (Figure 2c,d).

In contrast to the last decade of the 20th century, when the main progress in the field of oral implant research was made in alveolar bone resorption management to refine the different graft techniques [23], our findings show that in the 21st century there has been increasing interest in methods developed to overcome the grafting procedures and even a loss of interest in one of the most used augmentation techniques, *i.e.*, onlay bone grafting. It seems that dental implant scientific work is inspired more by the patient’s appraisals [24], seeking to improve minimally invasive surgical techniques [25], diminish patient morbidity, and shorten the treatment time. However, the role of industrial funding for conducting clinical studies should be taken into consideration. 32.4% of the clinical trials are supported by industry as a source of funding, which is a suitable way for companies not only to comply with safety and efficacy standards, but also to introduce their new products to the market [26]. This industry sponsorship may lead to biased reporting and pro-industry conclusions [27]. This does have the potential to reflect on ongoing trends in clinical research. In this connection for instance, the relatively innovative technique of guided implant surgery provides less painful and invasive treatment but at the same time is a more difficult and expensive procedure than conventional implant placement, demonstrating the same survival rate. However, a survey by Hof and coworkers [28] showed that the main priority for the patients when it comes to implant therapy remains the predictability of treatment success. The achievements brought about by ongoing clinical research, such as improved quality, ease of use of implant systems, as well as shorter treatment duration [29] may provide grounds for future researchers to face the challenge of preserving the perspectives of clinical implant research, and specifically, to enhance the relationship between private practice and science without involving marketing.

In order to adhere to ethical rules on explicit reporting, including also the disadvantages of any study, the researchers are obligated to report their study’s limitations. Undoubtedly, meta-analysis is the “gold standard” for performing any systematic review aiming at assessing treatment effects. Given that the variable investigated in the present study was the number of publications, the Poisson regression was selected as a statistical tool. The Poisson regression is used to model count data (in the present case this is the number of publications) and is an appropriate statistical method for predicting trends. Therefore, no methods estimating risk of bias, quality design, or heterogeneity of the studies provided by the meta-analysis were applied in this study.

A further limitation is presented by the use of only one database source. The findings in the present work are based on analysis, including studies from MEDLINE, and an additional hand search of six journals. However, the search strategy did not consider other databases such as EMBASE and the Cochrane Central Register of Controlled Trials.

In conclusion, the analysis of scientific literature on dental implants revealed several hot topics in the time period between 2001 and 2012. The most frequently covered surgical issues were bone substitutes (11.6%) and immediate implant placement, (7.5%), while the most prevalent prosthodontic topics involved immediate loading (14.3%) and cross-arch full bridges (8.0%). Given that the topics demonstrating the highest increase in interest were prosthodontic topics, *i.e.*, immediate loading (+6.3%) and platform switching (+2.9%), the interest in researching prosthodontic topics will most likely continue to increase.

## Figures and Tables

**Table 1:** Topics sorted by literature coverage. Absolute numbers of publications per year as well as the total percentage of all clinical papers 2001–2012 (* indicates prosthodontic topics).

Topic	2001	2002	2003	2004	2005	2006	2007	2008	2009	2010	2011	2012	Total
Immediate loading *	10	7	21	19	26	27	42	50	36	40	52	81	14.3%
Bone substitutes	13	18	20	16	24	20	15	30	33	44	53	46	11.6%
Cross-arch implant bridges *	13	10	24	20	16	11	11	22	20	20	25	39	8.0%
Immediate implant placement	7	10	9	13	7	7	23	21	21	30	30	38	7.5%
Simultaneous implant placement with augmentation	11	12	10	18	10	10	8	16	21	21	19	28	6.4%
Implant design	9	8	8	12	4	10	17	9	17	10	12	18	4.7%
Early loading *	6	8	12	9	14	8	13	17	6	14	8	14	4.5%
Onlay grafting	5	7	7	7	12	11	9	12	14	8	20	11	4.3%
Medically compr. patients	5	7	7	4	14	9	9	12	9	12	13	15	4.0%
Healing modality	6	10	9	7	11	8	8	14	10	9	7	7	3.7%
Transcrestal sinus floor elevation	2	5	2	3	7	5	5	9	10	10	11	18	3.0%
Implant diameter	4	2	3	8	2	6	6	6	7	10	6	17	2.7%
Flapless surgery	0	1	1	2	2	4	12	17	2	12	10	14	2.7%
Socket grafting	1	2	5	4	4	2	3	7	10	10	12	15	2.6%
Guided surgery	0	0	3	3	2	4	9	7	5	12	5	17	2.3%
Implant FPD-s	8	2	5	5	3	5	8	5	3	5	6	11	2.3%
Implant number	1	1	4	1	3	3	3	3	5	10	15	17	2.3%
Growth factors	2	0	4	2	6	2	5	7	12	9	7	8	2.2%
Implant length	2	0	2	5	7	4	5	5	4	6	6	15	2.1%
Peri-implantitis therapy	3	2	0	2	3	7	7	2	6	2	9	8	1.8%
Platform switching *	1	0	0	0	0	3	2	2	12	10	10	10	1.7%
Restorative materials *	0	3	1	2	2	4	2	6	3	4	6	10	1.5%
Tilted implants	1	0	0	0	3	0	4	3	3	7	8	10	1.4%
Abutment design *	4	1	3	2	0	1	4	2	5	3	10	3	1.3%
Smoking	0	3	1	4	2	3	2	3	3	2	4	4	1.1%
Piezoelectric surgery	0	0	0	0	1	0	2	4	1	8	2	7	0.9%
Early implant placement	0	1	0	2	2	1	0	3	3	1	2	4	0.7%
Cantiliver FPD-s *	0	0	1	3	0	2	1	1	1	0	2	4	0.5%
Cement *vs.* Screw retention *	0	0	0	2	0	3	0	2	1	0	2	3	0.5%
Ceramic implants	0	0	0	0	0	1	1	0	1	2	1	4	0.4%
Pterygoid implants	0	0	1	0	1	0	1	0	1	0	0	1	0.2%

**Table 2:** Topics demonstrating a significant increase (positive) or decrease (negative change) of scientific interest in the years 2001–2012.

Topic	2001–2004	2005–2008	2009–2012	*p*-value	Change
Immediate loading	57 (9.1%)	145 (16.2%)	209 (15.4%)	0.001	+6.3%
Platform switching	1 (0.2%)	7 (0.8%)	42 (3.1%)	0.001	+2.9%
Flapless implant surgery	4 (0.6%)	35 (3.9%)	38 (2.8%)	0.001	+2.2%
Guided implant surgery	6 (1.0%)	22 (2.5%)	39 (2.9%)	0.011	+1.9%
Growth factors	8 (1.3%)	20 (2.2%)	36 (2.7%)	0.014	+1.4%
Piezoelectric surgery	0 (0.0%)	7 (0.8%)	18 (1.3%)	0.015	+1.3%
Restorative materials	6 (1.0%)	14 (1.6%)	23 (1.7%)	0.011	+0.7%
Onlay grafting	26 (4.2%)	44 (4.9%)	53 (3.9%)	0.042	−0.3%
